# Chorusing, synchrony, and the evolutionary functions of rhythm

**DOI:** 10.3389/fpsyg.2014.01118

**Published:** 2014-10-10

**Authors:** Andrea Ravignani, Daniel L. Bowling, W. Tecumseh Fitch

**Affiliations:** Department of Cognitive Biology, Faculty of Life Sciences, University of Vienna, ViennaAustria

**Keywords:** rhythm, synchronization, isochrony, chorusing, evolution of communication, music perception, coupled oscillators, timing

## Abstract

A central goal of biomusicology is to understand the biological basis of human musicality. One approach to this problem has been to compare core components of human musicality (relative pitch perception, entrainment, etc.) with similar capacities in other animal species. Here we extend and clarify this comparative approach with respect to rhythm. First, whereas most comparisons between human music and animal acoustic behavior have focused on spectral properties (melody and harmony), we argue for the central importance of temporal properties, and propose that this domain is ripe for further comparative research. Second, whereas most rhythm research in non-human animals has examined animal timing in isolation, we consider how chorusing dynamics can shape individual timing, as in human music and dance, arguing that group behavior is key to understanding the adaptive functions of rhythm. To illustrate the interdependence between individual and chorusing dynamics, we present a computational model of chorusing agents relating individual call timing with synchronous group behavior. Third, we distinguish and clarify mechanistic and functional explanations of rhythmic phenomena, often conflated in the literature, arguing that this distinction is key for understanding the evolution of musicality. Fourth, we expand biomusicological discussions beyond the species typically considered, providing an overview of chorusing and rhythmic behavior across a broad range of taxa (orthopterans, fireflies, frogs, birds, and primates). Finally, we propose an “Evolving Signal Timing” hypothesis, suggesting that similarities between timing abilities in biological species will be based on comparable chorusing behaviors. We conclude that the comparative study of chorusing species can provide important insights into the adaptive function(s) of rhythmic behavior in our “proto-musical” primate ancestors, and thus inform our understanding of the biology and evolution of rhythm in human music and language.

## A COMPARATIVE MULTI-COMPONENT APPROACH TO THE EVOLUTION OF RHYTHM

An excellent starting point for a comparison of music with potentially proto-musical behaviors in other species is to adopt a “divide and conquer” strategy, avoiding a monolithic view of music, and squarely facing its composite, multi-component nature. A monolithic viewpoint leads naturally to unhelpful questions, such as “when did music evolve?” (as if this happened during one brief moment in human evolutionary history) or “where is music located in the brain?” (as if this complex cognitive ability occupies a single cortical region). The antidote to this monolithic conception is to recognize that any complex cognitive capability relies upon a suite of interacting cognitive capabilities. Each of these capabilities may have its own neural bases and independent evolutionary history.

In vision, for example, questions about motion or color perception may be meaningfully attacked at many levels of analysis (from molecules to brain circuits to evolution), but questions about “Vision”, conceived as an irreducible monolithic whole, cannot. We can ask and answer such questions as “when did human trichromatic color vision arise?” (answer: at the time of the catarrhine common ancestor of Old World Monkeys, apes, and humans, roughly 35 million years ago), or “what is the molecular basis of human trichromatic ability” (answer: a gene duplication of the “green” cone opsin, and subsequent divergence of its absorption peak; for documentation see [Regan et al., 2001; Vorobyev, 2004; Osorio and Vorobyev, 2008]).

But other questions about vision – more comparable to those typically asked about music – seem obviously wrong. Asking “when did vision evolve?” frames the problem in a misleading way from the outset. Vision has been evolving since our single-celled aquatic beginnings (rhodopsin was already present in our single-celled ancestors) continuously until the present, for roughly 1 billion years. Gains and losses of function, for many different adaptive reasons, have been happening throughout this long period. Because each component of vision has its own mechanistic and adaptive bases, a divide-and-conquer approach treating mechanistic components separately comes quite naturally to vision scientists (Gregory, 1970; Marr, 1982).

When considering rhythm in music, Lerdahl and Jackendoff (1983) and many subsequent commentators have correctly noted the need for such a breakdown (especially differentiating the ways in which musical rhythm differs from speech rhythm, see [Patel, 2003, 2006, 2008] for a discussion of similarities and differences). A clear distinction is required first between the perception and production of an isochronic pulse or *tactus*, typical of music but not of speech, and metrical structure (partially or entirely shared between speech and music [Liberman and Prince, 1977; Lerdahl and Jackendoff, 1983; Patel, 2008; Jackendoff, 2009]). A recent discussion of the importance of the pulse/meter distinction is (Fitch, 2013). Although we appreciate the importance of meter in the study of music, here we will focus on pulse (for discussion of the historical and cross-cultural significance of the pulse in human music see [Arom, 1991]). We will provide definitions and a categorization framework for quasi-periodic complex patterns that lack any strong/weak metrical characteristic, but extend the concept of pulse beyond metronomic isochronous sequences. Moreover, once we examine groups of individuals interacting there are multiple other important distinctions, as detailed below.

Our focus is on rhythmic comparisons between humans and other animals. With respect to human music, it is important to note that we are content to consider an idealized form in this paper. While musical reality and abstract representations of musical features have many important differences, we believe that an understanding of rhythm at a conceptual level provides a critical starting point for interspecies comparisons. In fact, the comparative approach is sometimes neglected for the obvious reason that non-human animals (“animals”, hereafter) lack systems equivalent to music and language in their human form. But, adopting the “divide and conquer” approach, it is equally clear that multiple “design features” of music and/or language are shared with other species. Most obviously, a capacity for complex vocal learning is required for both speech and song, and has evolved convergently in multiple animal lineages, although curiously not in non-human primates (Janik and Slater, 1997; Jarvis, 2004; Fitch, 2013). The relevance of vocal learning in animals, and birdsong in particular, for speech and song has been widely recognized for many years (Nottebohm, 1975; Marler, 2000; Fitch, 2013).

Unlike vocal learning, when it comes to rhythm there has been much less comparative research until recently. In the Descent of Man, Darwin (1871) argued that “The perception, if not the enjoyment, of musical cadences and of rhythm is probably common to all animals, and no doubt depends on the common physiological nature of their nervous systems”, later suggesting that “the instinctive power of producing musical notes and rhythms is developed low down in the animal series”. These comments received little empirical follow up, however, and Darwin never precisely characterized either “musical cadence” (his term for melody) or rhythm. Subsequent research suggests that Darwin may have been incorrect in these statements. Although further comparative work is required, evidence is mounting that even the capacity to entrain one’s own voice or movements to an externally generated pulse is quite limited in the animal kingdom (Patel, 2014).

## FUNCTION AND MECHANISM

Inquiry into human rhythmic capacities can be sharpened by adopting the approach of Niko Tinbergen, co-founder of modern ethology. Tinbergen distinguished four types of questions that can be asked about biological phenomenon: questions about *mechanism*, i.e., the neural underpinnings and causal chain of events that generate a behavior; questions about *ontogeny*, i.e., how a behavior develops over an organism’s lifespan; questions about *function,* i.e., a behavior’s adaptive role in the environment; and questions about *phylogeny,* the evolutionary history of a behavior (Tinbergen, 1963). Mechanistic and ontogenetic questions probe proximate causes, whereas functional and phylogenetic questions address ultimate causes. In the case of human rhythmic abilities, the mechanistic level has been studied most. Through detailed examination of motor behavior as well as EEG and fMRI data in rhythmic perception and production tasks, considerable progress toward understanding the neural bases of these phenomena has been made (Repp, 2005; Grahn and Brett, 2007; Grahn, 2009, 2012; Honing et al., 2012; Patel, 2013; Repp and Su, 2013). Also well-studied is the ontogenetic level, where questions about the development of rhythm perception have provided evidence that sensitivity to the metrical structure of rhythm may be present at birth (Winkler et al., 2009), and that sensitivity to the link between rhythm and movement is apparent in 7-month olds (Phillips-Silver and Trainor, 2005).

**FIGURE 1:**
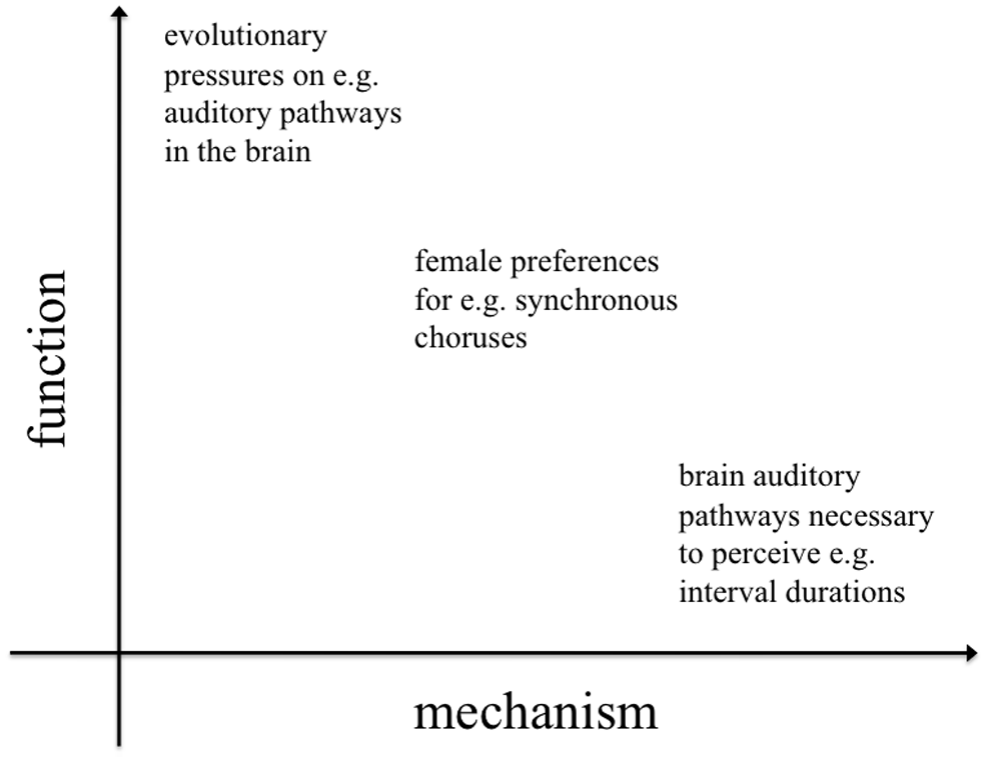
**Two-dimensional space representing levels of inquiry in evolution and behavior.** Asking why and how a given species exhibits proto-musical behaviors entails a number of more specific questions, whose answers might have a clear functional or mechanistic perspective or, sometimes, be an inextricable combination of both perspectives. While much of the previous literature has focused on the mechanisms underlying rhythm perception and production, the evolutionary functions of rhythm have received less attention.

Less well-studied, although this is beginning to change (Merker, 2000; Hagen and Bryant, 2003; Fitch, 2006; Hagen and Hammerstein, 2009; Dunbar, 2012; Bowling et al., 2013; Charlton, 2014), are the functional and phylogenetic levels of explanation. Despite a recent surge in interest, the ultimate causes of rhythmic capabilities (function and phylogeny) remain poorly understood and subject to the most speculation. Functional approaches investigate the evolutionary pressures that acted on early hominids, giving rise to entrainment and rhythm processing skills. Equally interesting are questions about the evolutionary history (phylogeny) of these skills. It is important to note that function and mechanism are not dichotomous; they represent different dimensions of explanation (addressing “why?” and “how?” questions respectively), but they often interact (**Figure 1**).

In the particular case of comparative research on rhythm (humans vs. non-human animals), most experiments have focused on proximate causes, asking questions like: Which rhythmic behaviors are present in newborns? How do these develop over the lifespan? What neural structures enable rhythm perception and auditory-motor synchronization? Do we share these neural mechanisms for rhythm with other species? Research aimed at answering mechanistic questions has focused on comparing birds and mammals (Patel et al., 2009a; Schachner et al., 2009; Zarco et al., 2009; Hasegawa et al., 2011; Honing et al., 2012; Cook et al., 2013; Hattori et al., 2013; Nagasaka et al., 2013; Ravignani et al., 2013a,b; Fuhrmann et al., 2014): sharing a common vertebrate ancestor implies many years of common evolutionary history, and the possibility that homologous brain structures are used to process rhythmic stimuli.

Research aimed at answering questions about ultimate causes (i.e., functional and phylogenetic questions) can benefit from considering rhythmic behaviors in a broader range of mostly neglected species. In particular, chorusing insects, synchronous frogs, and coordinated fireflies are often deemed unfit for human comparison on the basis of differences in nervous systems; synchronous behavior in insects, and anurans might be considered simple curiosities, worthy of little attention in research on rhythm in “higher vertebrates”. But neglecting insect and anuran species because the nervous mechanisms of synchronous behaviors are likely to be different from those of humans is to conflate mechanism and function. From a functional viewpoint, we argue that although the neural mechanisms may be different, similar rhythmic behaviors in different species may nonetheless have a similar function and evolutionary history, driven by similar selective pressures. The current review will thus highlight the relevance of such species in discussions of the evolution of rhythm.

## KEY CONCEPTS AND DEFINITIONS

### DEFINITIONS

Part of the difficulty in studying rhythm perception and production comes from a lack of clear, consistent terminology. The term “rhythm” itself, for example, is used in many different ways with interpretations ranging from simple (e.g., “the rhythm of the seasons”, which connotes nothing more than simple periodicity) to complex (e.g., the notion of rhythm and meter employed in Western music theory; Fitch, 2013). To address the problems that arise from terminological ambiguity, we propose a definitional framework that can be used to categorize temporal patterns for a comparative analysis of rhythm. The framework is sufficiently broad for almost any temporal pattern (i.e., any series of temporal intervals) produced by a single individual or group of individuals to fit within it. It consists of two separate hierarchies, one defining patterns that can be produced by a single individual (*the solo tree*) and one defining patterns that can be produced by multiple individuals (*the chorus tree)*.

Before further description, it is necessary to make two clarifications regarding the aim and scope of this framework. First, adopting a standard approach in animal behavior research, it categorizes patterns themselves, not the mechanisms responsible for their production. Questions about mechanism are of obvious importance (e.g., whether a mechanism is predictive or reactive; see “Mechanisms for Individual Timing”), but a detailed description of temporal signaling is necessary before they can be properly entertained. Accordingly, we make no assumptions about production mechanisms, and no claims regarding correspondence between specific mechanisms and particular categories. Second, as with any categorization scheme, its structure is determined by the properties we have selected as bases for categorization and it is thus only one of many possibilities (e.g., see [Merker, 2014]). Our aim is to provide a useful organization tool rather than the definitive structure. That being said, we believe the proposed categories are based on features of central importance in animal communication systems (including music).

#### The solo tree (Figure 2A)

**FIGURE 2:**
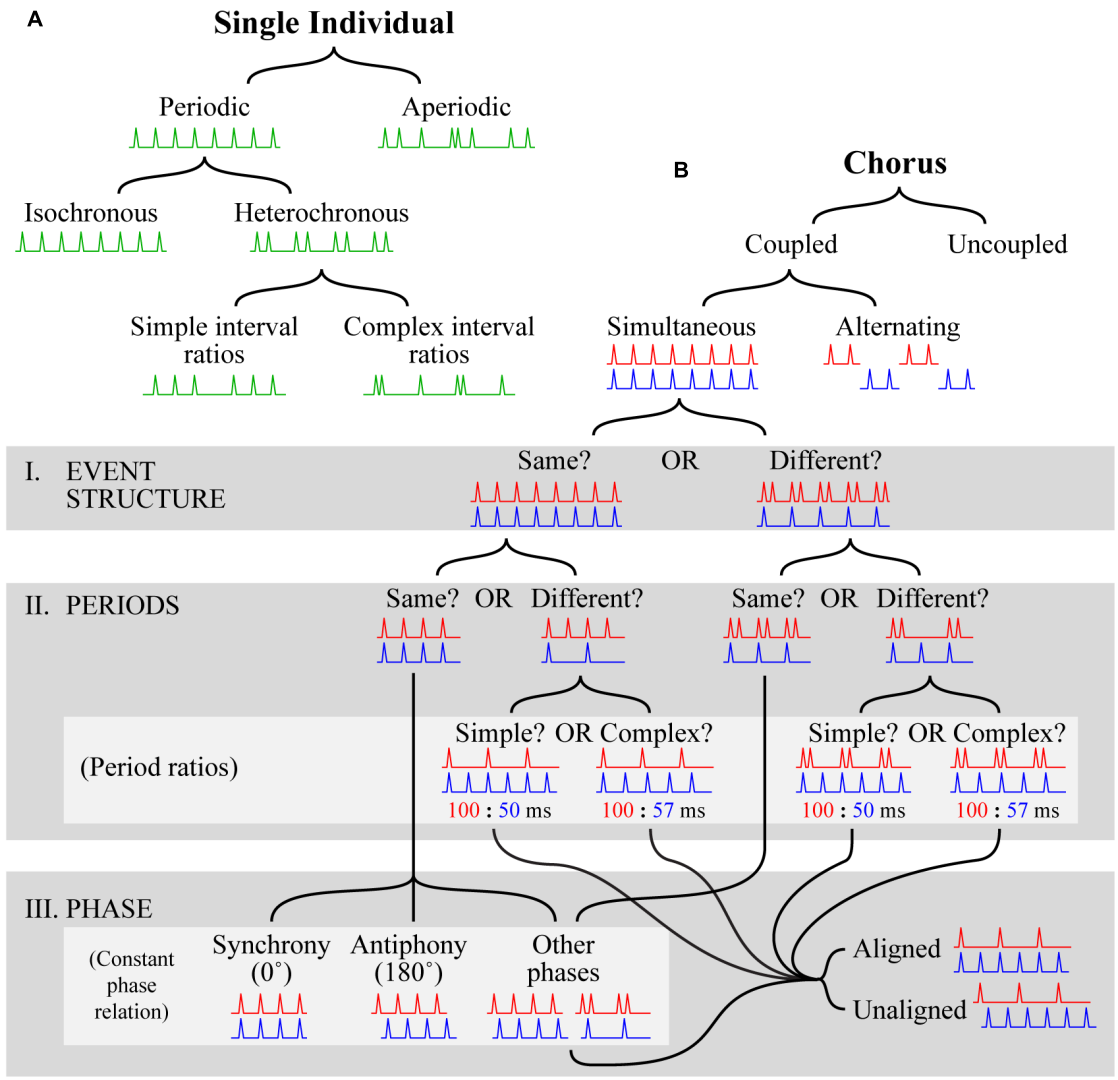
**Visual depiction of the proposed definitional framework. (A)** The *solo tree* hierarchically categorizes temporal patterns produced by a single individual. Categorization is accomplished by starting at the top and following the black lines down according to which branch provides a better fit at each level. Visual examples are shown in green at each level as a guide. Each example depicts a progression of events (spikes) in time (x-axis; left to right) that satisfies the conditions for inclusion in a particular category. For space reasons, the tree is only filled out for periodic patterns; for further description of aperiodic patterns see the main text. **(B)** The *chorus tree* hierarchically categorizes temporal patterns produced by multiple individuals. The format is the same as in **(A)**, with the exceptions that two patterns (red, displayed at the top, and blue, displayed at the bottom of each pattern pair) are necessary to show examples of category membership, gray boxes are used to highlight groups of categories (labeled according to the names following the roman numerals I–III), and light gray boxes are used to highlight subgroups of categories (labeled according to names in parentheses). In the light gray box labeled “period ratios”, the ratios given beneath each visual example relate the periods of the corresponding example patterns in milliseconds. For space reasons, the tree is only filled out for coupled simultaneous patterns; for further description of uncoupled choruses and coupled alternating choruses see the main text.

At the most fundamental level, a series of temporal intervals produced by a single individual can be *periodic* (i.e., regularly repeating) or *aperiodic* (i.e., non-repeating)^1^. Periodic patterns can be further divided into two categories, *isochronous*, and *heterochronous*. In isochronous patterns, the unit of repetition consists of only one interval (e.g., 1 s) repeated over and over again (e.g., 1 – 1 – 1 – 1 – 1 – 1 … ). In heterochronous patterns, the repeated unit consists of more than one interval. Heterochronous patterns can be further divided into two types, those where the different temporal intervals are related by ratios of relatively small integers (e.g., 1 – ½ – ½ – 1 – ½ – ½ – 1 …; *simple interval ratios*), and those where larger integers are required (e.g., 1 – 13/23 – 17/26 – 1 – 13/23 – 17/26 – 1 …; *complex interval ratios*). In principle, the distinction between which ratios are simple and which are complex is somewhat arbitrary, but in practice this distinction is made clear by musical rhythms, which typically comprise ratios such as ^1^/_2_, ^1^/_3_, ^1^/_4_, ^1^/_8_, and ^3^/_4_, but avoid ratios like ^13^/_23_. The distinction between simple and complex interval ratios can also be applied to aperiodic patterns (not shown in **Figure 2A**), the difference being that such patterns do not repeat.

#### The chorus tree (Figure 2B)

The situation quickly becomes more complex when two or more signalers are involved. Perhaps the most fundamental distinction concerning groups of signalers is whether or not individuals exert a causal influence on each other, i.e., whether they are *coupled* or *uncoupled.* We focus on coupled chorusing here because it implies causal interaction between individuals and is thus characteristic of the animal communication systems under consideration. Uncoupled chorusing occurs when individuals in some form of group (determined, for example, by spatial proximity) generate signals without causal relationships (e.g., the different conversations at a cocktail party). Arbitrary temporal relations also exist between individuals who, despite some spatial proximity, do not constitute a group (e.g., two pianists practicing in separate rooms of a conservatory), but referring to such situations as “choruses” seems inappropriate.

Returning to coupled choruses, the next distinction is whether the individual patterns are produced at the same time (*simultaneous*), or one after the other with little or no delay (*alternation*). This is particularly important in duetting species (including a wide variety of bird species and some primates), where individual signals may or may not overlap (see sections on “Beyond Insects and Anurans: Additional Insights?” and “Synchrony and Antiphonal Chorusing in Birds” below). We will first cover the portion of the tree representing simultaneous chorusing in detail, and then suggest how similar logic can be applied to alternation chorusing (not shown in **Figure 2B**). Regarding simultaneous chorusing, the remaining distinctions can be sorted into three groups. First, we ask whether the *event structure* of the component patterns is the same or different (**Figure 2B**, I). The event structure of two patterns is the same if the sequences of temporal intervals are the same, or related by a rational number multiplier; otherwise, the event structure is different. For example, two patterns, one isochronous and the other heterochronous, exhibit a different event structure (**Figure 2B**, I. EVENT STRUCTURE: “Different”). Second, we ask whether the *periods* of the component patterns are the same or different (**Figure 2B**, II). If the periods are different, a useful sub-distinction is whether they are related by ratios of relatively small integers (e.g., ^1^/_2_, ^1^/_3_, ^1^/_4_, ^1^/_8_, ^3^/_4_; *simple period ratios*) or whether larger integers are required (e.g., ^2^/_15_, ^13^/_23_, ^17^/_26_; *complex period ratios*). Third, we examine the *phase relationship* between component patterns (**Figure 2B**, III). If patterns have the same event structure, and the same period, they will have a *constant phase relation*, meaning that their phases will be related by a constant phase offset. Although any offset is possible, two have been given special names: *synchrony* refers to a phase offset of 0^∘^ (i.e., individuals produce events at the same times); and *antiphony* refers to a phase offset of 180^∘^ (i.e., individuals alternate). If patterns have different event structures, but the same period, they will also exhibit a constant phase relation, but the concepts of synchrony and antiphony do not apply. The phase of the patterns may still be related by 0 or 180^∘^ of course, but the fact that only some events will co-occur (or anti-occur) rules out precise usage of these terms (as defined here). Regardless of whether their event structure is the same or different, if patterns have different periods their phase relationship will continually cycle from 0 to 359^∘^. It is thus more useful to think about their phase as being *aligned*, with many events co-occurring, or *unaligned,* with few if any events co-occurring. The aligned vs. unaligned distinction can also be usefully applied to patterns with different event structure but the same period.

The proposed distinctions regarding event structure and period can also be applied to alternation chorusing (in precisely the same way). The distinctions regarding phase relationships are slightly different, however, and should be aimed at distinguishing choruses with overlapping parts from those with non-overlapping parts, as well as characterizing the extent of corresponding overlap or delay. Another important consideration is the potential equivalence between alternation chorusing and simultaneous antiphonal chorusing. We propose that a useful distinction in differentiating these admittedly similar forms, concerns the complexity of the alternating parts. In alternation chorusing, the alternating parts can be quite complex, consisting of multiple events, heterochronously arranged (e.g., the duets of plain tailed wrens and some gibbon species; see sections on “Beyond Insects and Anurans: Additional Insights?” and “Synchrony and Antiphonal Chorusing in Birds” below). In contrast, the alternating parts in simultaneous antiphonal chorusing are much simpler, typically consisting of only one event (e.g., *Mecopoda* species S; see section “More Than Isochrony: *Mecopoda*, A Multifaceted Rhythmic Insect” below). Examples detailing how this framework can be used to categorize some of the temporal patterns produced by species reviewed in this paper are given in **Table 1**.

**Table 1:** Application of the proposed definitional framework to a selection of the reviewed species.

Species	Description of chorusing behavior
*Pteropyx malaccae* (Indomalayan firefly; Buck, 1938)	• Coupled, simultaneous chorus of individuals producing the same, isochronous event structures, with the same periods, and synchronous phase.
*Neoconocephalus spiza* (Neotropical katydid; Greenfield and Roizen, 1993)	• Coupled, simultaneous chorus of individuals producing the same, isochronous event structure, with the same periods, and a constant phase relation (typically small).
*Kassina fusca* (Afrotropical frog; Grafe, 1999)	• Coupled, simultaneous chorus of individuals producing the same, isochronous event structure, with the same periods, and a constant phase relation (small when signaling with conspecifics, large when signaling with heterospecifics).
*Mecopoda* species S (Indomalayan katydid; Sismondo, 1990)	• Coupled, simultaneous chorus of individuals producing the same, isochronous event structure, with the same or different periods. • When periods are the same, phase is typically synchronous or antiphonal. • When periods are different, they are typically related by simple period ratios, and phase is aligned.
*Thryothorus euophrys* (Neotropical plain-tailed wren; Mann et al., 2006)	• Within-sex: coupled, simultaneous chorus of individuals producing the same, heterochronous event structure, with the same periods, and synchronous phase. • Between-sexes: coupled, alternating chorus of individuals producing different, heterochronous event structures, with different periods.
Human music	• Greatest diversity of forms. • Coupled, simultaneous and/or alternating chorus of individuals producing the same and/or different, isochronous and/or heterochronous event structures. • A typical example is a coupled, simultaneous chorus of individuals producing different, isochronous and heterochronous event structures, with different periods, related by simple period ratios, and with aligned phase.

*The left column lists the name of a species in italics, a common descriptor in parentheses, and the reference in which corresponding chorusing behavior is described. The right column describes the chorusing behavior of each species using the definitional framework proposed in “Definitions” section (see also **Figure 2A**). Note that we do not claim to exhaustively catalog the chorusing capacity of each species presented in this table. Our aim is rather to describe published chorusing data in a clear and consistent fashion.*

### MECHANISMS FOR INDIVIDUAL TIMING

What kind(s) of neural mechanisms does an organism need in order to process rhythmic sequences and adjust its behavior to others’ in a related fashion? Although this paper focuses on the function of rhythm, we briefly consider current opinions concerning the mechanisms underlying rhythmic behavior.

A number of mechanisms have been proposed to explain human timing abilities. A first division is between “explicit” and “implicit” models. Explicit models are based on an accumulator that explicitly keeps track of discrete temporal units, and then compares the total to some quantity stored in memory (e.g., Matell and Meck, 2000; Allman et al., 2014). Implicit models instead employ a dynamical systems approach, suggesting that timing abilities are based on neural oscillations, which among other things can couple to and resonate with external auditory signals (Large, 2008). Another class of timing models proposes compromises between explicit and implicit approaches, whereas still others depart from these ideas entirely (for an overview, see [Mauk and Buonomano, 2004; Allman et al., 2014]).

A second important distinction in the study of timing mechanisms is drawn between predictive and reactive timing. An organism using a predictive timing mechanism will use past information to generate some form of internal model capable of anticipating the timing of future events (e.g., a human tapping along to a song). In contrast, an organism using a reactive timing mechanism does not anticipate future events, and instead times its behavior by responding to external stimulation with some delay (which may be flexible and is often dependent on the nature of the stimulus). For instance, some frogs reactively time their calls, so as to partially overlap or clearly follow the external stimulus depending on its acoustic properties (Grafe, 1999; see section “Leaders and Followers in Context-Timed Signaling” below). Although the prediction/reaction distinction is not the focus of this paper, we recognize its importance also for functional questions. Reactive timing mechanisms are typically considered to be simpler than predictive mechanisms, but their nuanced function in some species has the potential to inform on the functional origins of human rhythm. Most animal comparative research in this area has, until now, focused on the (rare) instances of predictive timing. We will present several previously neglected cases of reactive or “ambiguous” timing (a term used to describe situations where mechanisms are particularly opaque) to reevaluate their importance.

The richest source of evidence concerning the evolutionary history of rhythmic capacities is broad interspecies comparisons. In making such comparisons, it is useful to have precise definitions of rhythmic behaviors that apply across species (which we have attempted to provide above), data on individual timing and chorusing dynamics in multiple species, and some general knowledge regarding the potential evolutionary functions of timed signaling independent of any particular species. In light of the definitions provided in the previous section (see **Figure 2**), we now review the empirical literature on chorusing in a broad range of species, and survey the hypotheses regarding evolutionary function.

## TIMING ACROSS SPECIES AND EVOLUTIONARY HYPOTHESES

### SYNCHRONOUS FIREFLIES

The most prominent examples of animal entrainment do not come from birds and mammals, but rather from insects and frogs. A remarkable example of massive group synchrony is found in several species of firefly (Buck, 1938). Fireflies are winged beetles in the family Lampyridae, which contains roughly 2000 species. Fireflies have a capacity for bioluminescence, often used in a courtship and mating context, sometimes by both sexes but often by males alone. In several firefly species, e.g., the Indomalayan *Pteroptyx malaccae*, large groups of males entrain, such that all the individuals in a tree flash in almost perfect 0^∘^ synchrony (Buck and Buck, 1968). This level of synchronization is outstanding among non-human species, and *P. malaccae* probably represents the organism in which the precision of synchronization abilities most closely compares to those exhibited in human ensemble music. The neural and physical basis for firefly synchrony has been modeled (Ermentrout, 1991; Strogatz and Stewart, 1993; Strogatz, 2003), and it seems relatively clear that the tight synchronization of flashing in this species requires accelerating or decelerating an internal isochronous rhythm (pulse tempo adjustment) and adjusting the phase of this “inner clock” (phase alignment). This combination appears very unusual: most animals, if they can entrain at all, only do so to a narrow range of fixed tempos. Firefly entrainment closely matches what a human listener must do in order to clap along or dance to a novel piece of music.

Surprisingly, despite many decades of study of these fireflies (Buck, 1938, 1988), the evolutionary function of *Pteroptyx* synchronous flashing remains uncertain (Greenfield, 2005). One hypothesis is that synchronization acts to sum signals together, creating a more powerful overall signal to attract females from further away. Such synchronization might be considered to be a cooperative endeavor, where, by combining their relatively weak signals, a group of males can collectively generate a brighter signal. This in turn would alert and attract females from further away (cf. the “beacon hypothesis” Merker, 2000). However, that individual flashes sum and attract more females has never been demonstrated empirically in any species, and it remains uncertain whether the net number of mates attracted per male is in fact increased by synchronous flashing. Data from other species (e.g., several frog species, [Gerhardt and Huber, 2002]) suggests that, although females do prefer choruses over single males, and larger choruses over smaller ones, this female preference is *not* strong enough to compensate for the dilution in sex ratio caused by the larger number of competing males (see [Ryan et al., 1981] for an exception). By analogy, if a rock band attracts more potential mates than a soloist, but the singer, drummer and guitar player get all the girls, what’s in it for the bass player? Such questions have led researchers to propose other adaptive explanations, several of which have better empirical support than the summation hypothesis.

### BENEFITS OF CHORUSING BEHAVIOR

In the auditory domain, quite a few frog species are known in which males mating calls are reasonably well-synchronized (Wells, 1977), and in many insect species where males call to attract females, including cicadas and many orthopterans, spontaneous entrainment of these calls is observed to produce large, roughly synchronized choruses of calling males (Alexander, 1975). These acoustic displays rarely, or never, approach the degree of synchronization seen in *P. malaccae* (Gerhardt and Huber, 2002; Greenfield, 2005).

Does signaling in a chorus actually confer a reproductive advantage to individual signalers as the beacon hypothesis suggests? Experiments on meadow crickets seem to suggest that it does. Females “asked” to choose between equal-amplitude recordings of a single cricket and a male cricket duet preferred the latter (Morris et al., 1978). Hence, individual males might increase their opportunities to mate by signaling in groups (Merker, 2000; Merker et al., 2009). Other possible benefits of chorusing behavior are rather diverse. For instance, rattan ants (genus: *Camponotus*) are capable of generating a startling “rattle” by locally synchronizing their movements to shake the vines they inhabit (Merker et al., 2009). This response is triggered when an inhabited vine is touched and may serve to deter predators. Similarly, frogs may use chorusing to confuse predator’s auditory localization abilities, thus “hiding in the crowd” (Tuttle and Ryan, 1982). Additional examples and a brief discussion of their significance for human music can be found in Merker et al. (2009).

### SYNCHRONY AS AN EPIPHENOMENON OF COMPETITION

In at least some species, e.g., the Neotropical katydid *Neoconocephalus spiza* (order Orthoptera, family *Tettigoniidae*), it now seems clear that synchronization is a non-adaptive by-product of competitive interactions, resulting from males attempting to “jam” each others’ signal (Greenfield and Roizen, 1993). In this case, rather than inferring a general pulse and adjusting its phase, males very rapidly (e.g., 40–50 ms reaction times) react to a neighbor’s individual pulses. This reactive male can then produce his own output after a slight lag (leading to a staggered overlap of calls), or alternatively adjust his call to coincide with, but slightly lead, the other males. This leads to a leap-frog phenomenon, in which males roughly alternate in leading and following roles. Since females in many species appear to be preferentially attracted to the leading male, synchrony in these cases is likely to be epiphenomenal: the real causal agent is a competitive battle for slight temporal primacy. Thus, these katydids end up synchronized although each individual attempts to enhance its own conspicuousness by leading.

### LEADERS AND FOLLOWERS IN CONTEXT-TIMED SIGNALING

Other species appear to actively seek some form of constant phase relation. For instance, male frogs of the Afrotropical species *Kassina fusca* (order Anura, family *Hyperoliidae*) acoustically compete for females, trying to win themselves “broadcasting time” against both conspecifics and heterospecifics (Grafe, 1999). Male calls partially overlap in time: what drives this overlap, and what function does it serve? To answer these questions, field research has explored both sides of a possible sexual selection mechanism: (i) male frogs’ flexibility to overlap, dependent on the type of stimulus presented, and (ii) which male caller is preferred by females in a series of overlapping sounds (Grafe, 1999). Playback experiments show that overlapping calls are not a mere byproduct of frogs starting to vocalize at random times. Instead, males flexibly adjust their call onsets. If prompted with recorded conspecific calls, a male times its call to start during the second half of the played call (i.e., a small phase delay). Calls of other frog species (or even white noise), however, elicit a call with a larger phase delay, showing that in this context the male waits until the stimulus has ended to broadcast its call (Grafe, 1999). This differential response to conspecific calls vs. all other sounds of different length also shows that the timed responses can be flexibly adapted according to the temporal and spectral properties of acoustic context. The frogs exert some form of rhythmic control over their calls following a simple rule (“hear a conspecific?”: overlap by 50% or less; “hear anything else?”: wait for offset).

What is the evolutionary function of *K. fusca’s* chorusing behavior? Playback experiments suggest that it reflects female preferences: If a female is presented with two recorded chirps coming from two loudspeakers at different times, she will preferentially approach the second call in a sequence of two if they overlap by 10–25%, but switch to the first if the two calls overlap almost completely (Grafe, 1999). Considering that the call overlap under natural conditions is short (although greater than 0%), females have a natural preference for followers rather than chorus leaders, putting pressure on males to overlap, but not fully. Thus, it seems that at least part of the evolutionary function of chorusing behavior in *K. fusca* is to attract females (although not necessarily through sexual selection, cf. Ryan, 1998; Grafe, 1999).

### MORE THAN ISOCHRONY: *MECOPODA*, A MULTIFACETED RHYTHMIC INSECT

*Mecopoda* species S (family *Tettigoniidae*; most likely *Mecopoda elongata* but referred to as “species S” by Sismondo [1990], Hartbauer et al. [2005]) is a species of Indomalayan katydid with particular versatility in timing calls in response to conspecifics. These insects have attracted considerable attention for exhibiting abrupt changes in call frequencies, generating entrainment patterns that depend on the distance between individuals (Sismondo, 1990).

Imagine a field full of katydids. Walk through the field and choose a specific “reference katydid”, maybe one with a pitch slightly lower than the others. As you compare its chirp timing with the others’, you will notice that, within a few meters’ range, all the katydids chirp in synchrony with the reference, and with little variation from an isochronous pattern. But, if you walk further away, katydids begin to switch from synchrony to antiphony, and at an overall slower call rate (Sismondo, 1990). Inspired by observations of this flexible chorusing, playback experiments have shown that pairs of katydids, each producing isochronous patterns, can result in choruses of different periods related by simple ratios (an analogy from human music would be one voice playing two beats, while the other plays a triplet, i.e., 2:3). Mechanistically this seems to be because the neural phase-resetting mechanism in these katydids responds to differences in sound intensity. Thus, different chorusing regimes (e.g., synchrony, antiphony, and simple period ratios) are the only mathematical solutions to the system (Sismondo, 1990). This kind of “inevitable chorusing outcome” matches the behavior of abstract systems of physical oscillators surprisingly well (Kuramoto, 1975; Strogatz, 2000, 2003).

### GEOMETRY FOR A SYNCHRONOUS CHORUS

A central adaptive question concerning chorusing is: does it pay to be part of a chorus? In other words, will an individual organism increase its fitness, on average, by signaling as part of a group? As previously discussed, joining a chorus might allow a male to mate with more females, or decrease the risk of predation (allowing your neighbor to be eaten instead of you). On the contrary, chorusing may be detrimental by diluting mating opportunities, and increasing predation risk by attracting not only more mates, but also more predators. The costs and benefits of group chorusing can be investigated empirically by examining how many individuals call together (within hearing range), how many are killed by predators, and how many females are attracted by choruses of different sizes. Ryan et al. (1981) examined these factors in the neotropical frog, *Physalaemus pustulosus.* They found that chorus size (the number of individuals in a chorus) is negatively correlated with the probability of an individual being eaten by predators, despite not being correlated with the overall predation rate. This suggests that joining a chorus can increase fitness by decreasing the risk of being eaten. They also found that chorus size is positively correlated with the average number of females per male, suggesting that another advantage of joining a chorus might be increased mating opportunities. Both of these findings also suggest that it is better to join larger choruses than smaller ones. It thus appears that chorusing behavior serves several distinct evolutionary functions in this species. We know very little, however, about how such potential functions are related to the detailed temporal characteristics of chorusing behavior like synchrony.

Some of the ideas underlying the costs and benefits of group membership were originally formalized by Hamilton (1971), who proposed a model of spatial clustering in non-human animals (although his model did not consider calls or chorusing). Hamilton’s “herd” model featured frogs randomly distributed over a circular pond, under constant threat of predation by a snake. Empty space surrounding individuals makes them vulnerable. To reduce its conspicuousness to predators, each frog would tend to jump in-between two adjacent frogs. Hamilton’s model offers one explanation of why large aggregations of certain animals form in space. Given the work of Ryan et al. (1981) establishing other benefits of being in close spatial proximity (i.e., increased mates and decreased predation), can Hamilton’s herd model also explain clustering tendencies in call timing and rhythmic patterns for chorusing behavior?

**FIGURE 3:**
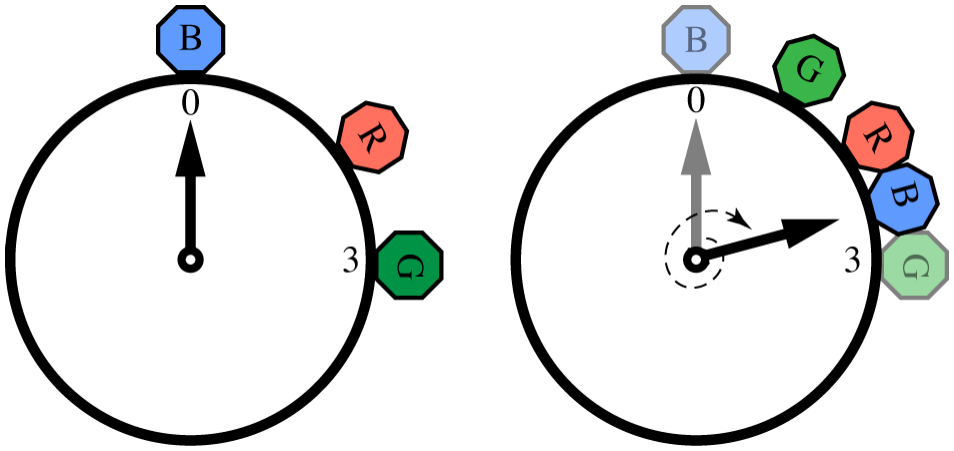
**Calling onsets of 3 individuals over two time periods, following a “selfish chorusing” phase-shift rule.** In the first period (left) individual B (blue) is surrounded by much silence, hence in the second period (right) B will postpone its call and signal between R (red) and G (green). Likewise, G will anticipate its call and signal between B and R. Individual R’s optimal strategy is to keep its call onset unchanged. Figure reproduced and modified from (Ravignani, 2014).

Ravignani (2014) adapted the herd mathematical framework to model call onsets and durations within a chorus. Adopting the metaphor of a clock to represent events that repeat in time, the onset of each individual call can be thought of as a specific phase angle in a circular space (**Figure 3**). Now, instead of each individual varying its location in space (as in the herd model), a caller shifts the temporal “location” of its call onset, depending on the location of other individuals’ calls. Just as the jumping frogs of the herd model shunned empty space in favor of crowded space, callers avoid silent phase regions in favor of temporal clustering.

**FIGURE 4:**
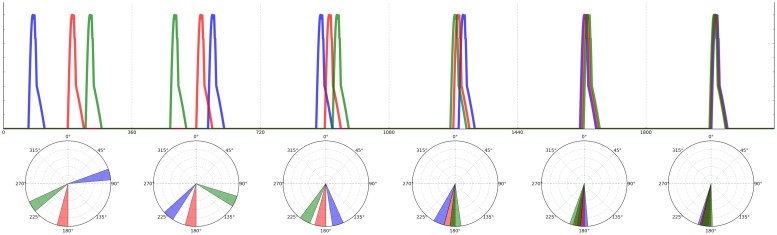
**Agent based simulation of 3 individuals calling over six time periods (based on the model in Ravignani, 2014).** Phases’ onsets of calls are depicted on top as time series. Time periods are separated by vertical lines, with the equivalent polar representation below each period. During the first time period, individuals listen to each other, so to shift their calls’ onset in the second period, repeating this strategy over time. The blue and green individuals will alternate as leader and follower over time periods; the red individual’s best strategy is, in this example, to keep its call onset constant over time periods. The agents will have reached almost perfect synchrony by period 6.

This acoustic adaptation of the herd model makes a clear prediction: for a broad range of initial conditions, all individuals will end up calling roughly in synchrony (and possibly also isochronously; Ravignani, 2014). However, as Hamilton (1971) noticed in his original model, there are some initial conditions that prevent aggregation, which also apply acoustically. In particular, if there are N individuals each calling at 2πi/N radians (*i* = 1,...,N), the system will become locked in a state of alternation, where individuals continuously “change their minds” about the ideal time for their call onsets and produce non-isochronous, although globally repeating patterns. This outcome can also be observed in simple physical systems of coupled oscillators (Kuramoto, 1975; Strogatz, 2000, 2003), where a statistical steady-state can be achieved in which the overall global distribution of phases is constant over time, even though each oscillator shows no consistent phase relations with the others. The results of a computer simulation of the acoustic herd model showing the evolution of chorusing over time for a small population of individuals are shown in **Figure 4** (Ravignani, 2014). In such simulations, the state of synchrony is achieved very quickly, usually requiring only 5–10 time periods (arbitrary units). This computational model is further supported by recent research in non-human primates (Nagasaka et al., 2013; Ravignani et al., 2013b; Fuhrmann et al., 2014). Together, these findings highlight the importance of taking into account group behavior and social factors in the study of individual animal timing.

### BEYOND INSECTS AND ANURANS: ADDITIONAL INSIGHTS?

Why have frogs and insects been so neglected until now in the study of human rhythm? Three factors appear particularly important in differentiating the behavior of these animals from rhythmic behavior in humans: complexity, flexibility and modality (Patel et al., 2009b). Because frogs and insects have relatively small nervous systems (and less entrainment flexibility than humans), the mechanisms underlying frog and insect chorusing behavior are assumed to be quite dissimilar to those that govern human rhythmic abilities in music (Patel, 2006). Frog and insect timing mechanisms are also likely to be primarily (if not exclusively) modality-specific, and are probably underpinned by relatively inflexible neural circuitry (Patel, 2006). The time scales of signaling vary greatly across the species considered above. Some call slowly, e.g., *K. fusca* signaling once every 2–8 s (Grafe, 1999), while others naturally signal much faster, e.g., *Mecopoda* Species S signals once every 1–3 s (especially at high temperatures; Sismondo, 1990). Such period differences may correlate with (or be causally related to) differences in function and mechanism across species. For more extensive reviews on time perception in insects and anurans see (Hartbauer and Römer, 2014; Rose, 2014). These systems are also strictly isochronic at an individual level, while musical rhythms are typically heterochronous. Although much dance music has a perfectly even pulse at the musical surface (e.g., a “four on the floor” bass drum part), it is far more typical to have patterns in which some notes of the underlying pulse are *not* sounded, and where many additional notes are interspersed between pulses. This makes even the simplest aspect of human rhythm – the inference of a steady pulse from a complex musical surface – go beyond the insect and frog examples considered here in terms of cognitive complexity (cf. Fitch, 2012). Thus, while the existence of numerous synchronizing species provides comparative evidence regarding adaptive hypotheses about the evolution of entrainment (cf. Alexander, 1975; Wells, 1977; Greenfield, 1994; Gerhardt and Huber, 2002), these species may teach us less about the neural circuitry underlying human rhythmic abilities. In particular, a prominent hypothesis connects vocal learning in humans and some other animal species with the ability to perceive and entrain to a regular pulse (Patel, 2006). Under this hypothesis, the ability to learn vocalizations from auditory stimulation implies a tight coupling between the neural circuits that govern audition and motor behavior, and this coupling is considered necessary (although not sufficient) for human-like entrainment abilities (Patel, 2006). Specifically, advocates of the vocal learning hypothesis propose that periodic movements may be “rehearsed” in motor regions of the brain, allowing individuals to better predict the timing of future regular pulses (Patel and Iversen, 2014).

Early work connecting animal behavior with musical rhythm pointed to the absence of reports of spontaneous animal entrainment to music (Patel, 2006). Since then, a few species have been tested in the lab and shown capable of limited or full entrainment capabilities (for reviews see [Fitch, 2013; Ravignani et al., 2013a; Repp and Su, 2013]). A number of parrot species (Patel et al., 2009a; Schachner et al., 2009; Hasegawa et al., 2011) and one sea lion (Cook et al., 2013) have been shown to be capable of synchronizing to an external pulse under different tempi. Recent evidence in macaques (Zarco et al., 2009; Honing et al., 2012; Nagasaka et al., 2013) and one chimpanzee (Hattori et al., 2013) suggests that other primates might share only a subset of the critical components necessary for rhythmic entrainment with humans, at least when tested in the lab. A recent hypothesis proposes, in fact, that while single-interval timing is shared among primates (including humans) due to common ancestry, beat-based timing (enabling rhythmic entrainment) is a human idiosyncrasy among primates (Merchant and Honing, 2013; for further consideration of interval and beat-based timing, see also Teki et al., 2011).

The study of relatively simple species has proved a fertile source of functional hypotheses regarding the adaptive function(s) of chorusing behavior. We now turn to studies of natural group calling behavior in birds and mammals.

### SYNCHRONY AND ANTIPHONAL CHORUSING IN BIRDS

A well-known hypothesis in animal communication is that the evolution of large vocal repertoires may be driven by sexual selection, especially in birds (Slater, 2000). This idea appears to apply less to the individual elements in a song than to the degree of combinatorial variety (Slater, 1981). However, many similarities that exist between birdsong and music are likely to be a result of evolutionary convergence rather than homology (Slater, 2000) since our last common ancestor with birds lived roughly 300 million years ago. We hence caution against drawing *mechanistic* parallels between birdsong and music, which are more likely to be analogous than homologous behaviors. Nevertheless, despite a lack of mechanistic or even phenomenological similarity, research asking why vocal display evolved in birds (and other vocal learners) can inform us about general evolutionary pressures that may have also shaped human music.

Duetting, defined as coupled simultaneous and/or alternating chorusing, occurs in approximately 400 bird species, covering approximately 40% of bird families (Hall, 2009). Some of these examples might involve random (i.e., uncoupled) interactions leading to spuriously interdependent choruses (e.g., similar event structure or simple period ratios arising by chance) rather than concerted group behavior. Hence, adequate null models for chorusing behavior are essential to distinguish random from interactive group behavior (Kuramoto, 1975; Strogatz and Stewart, 1993). Considering the tremendous variety that exists in birdsong, at least some coincidental similarity to some human music seems almost inevitable (Slater, 2000). Importantly, a periodic rhythm may simply be produced by “a mechanism operating at its resonant frequency” (Slater, 2000) following the principle of energy minimization, and therefore does not require more elaborate explanations in terms of functional “design”. At least for some species, however, the complex intertwining of note onsets and melodic lines goes beyond this base case.

The chorusing behavior of Neotropical plain-tailed wrens (*Thryothorus* = *Pheugopedius euophrys*)*;* order Passeriformes, family Troglodytidae) is particularly interesting in this respect. In this species, the number of birds chorusing at one time is relatively high, with groups typically comprised of four individuals (two-bird duets also occur, but are the exception). A single wren chorus contains both synchronous and antiphonal features (Mann et al., 2006); the presence of both features is quite rare in other avian species. In particular, male and female wrens alternate their parts within a chorus, while members of the same sex show nearly perfect synchrony of the same phrases (Mann et al., 2006). From a mechanistic perspective we do not know much about wrens’ individual timing: At one hypothetical extreme, each bird could finely adjust its call onset based on predictive timing, but it could also be that each bird knows the whole song template and “unmutes” itself depending on the part without the need of a fine timing neural structure (Merker et al., 2009). Despite our lack of knowledge regarding mechanisms in this context, the mapping between sex and chorus timing raises fascinating questions about the evolutionary functions of rhythm in this species. Female–male alternation in choruses is often associated with signaling a strong pair-bond in defense of territories and/or resources (as with gibbons; see below). Male–male synchrony, as seen in orthopterans and fireflies, seems to relate to female preferences. As no single function seems to explain both the alternating and synchronous features of wren chorusing, a parsimonious explanation involves multiple evolutionary pressures. The impressive coordinated behavior of plain-tailed wrens may represent the interaction between sexual advertising and group territorial dynamics. This may also be the case for rhythmic abilities in human music.

### CHORUSING AND SYNCHRONY IN PRIMATES

Turning to primates, chorusing behavior, in the form of duetting, has been observed in at least 4 unrelated genera: *Indri*, *Tarsius*, *Presbytis*, and *Hylobates* (Haimoff, 1986). As these primate species are not closely related, duetting probably evolved independently in each of these groups. The indri (*Indris indris*) is a lemur species that engages in duetting behavior (Giacoma et al., 2010), and the extent to which indris actively seek synchrony is currently being investigated (Gamba et al., 2014). The best studied duetting species are various gibbons or “lesser apes”, in which song repertoires are genetically inherited, rather than learned as in songbirds (Geissmann, 2000). Mated pairs of gibbons sing duets in which the repertoire of both individuals is temporally tightly coordinated. Females also produce solos known as “great calls” that comprise a series of long, gender-specific calls increasing in tempo. As with plain-tailed wrens, singing in male and female gibbons may be the result of multiple sex-specific selection pressures. The extent to which gibbons exert voluntary control over their call timing is unclear. While gibbon duets clearly feature temporal coordination, they more closely resemble conversational turn-taking than polyphonic musical duets with an underlying regular beat. Mated pairs of siamangs (*Symphalangus syndactylus*; a type of gibbon) do perform simultaneous “barks” during the “warm-up” phase of their duets, but these appear to exhibit a phase delay, implying a kind of reactive “shadowing”, rather than joint adherence to a common pulse (unpublished video analysis, Bjorn Merker, personal communication). Functionally, gibbon song has been hypothesized to play a role in territorial defense and pair-bonding (possibly through costly learning of synchronization), and it does not seem that songs are used to attract mates (Geissmann, 2000).

Apart from the four genera mentioned above, some additional primate species are worth mentioning. Bonobos have been reported to perform a kind of “staccato hooting” in both captive and wild settings that may follow an approximate 2 Hz isochronous pulse (De Waal, 1988; Bermejo and Omedes, 2000; Merker et al., 2009). However, some caution is warranted here given that none of these studies actually measured timing in a rigorous way. Nevertheless, if these claims can be substantiated by experimental evidence, bonobo staccato hooting would be extremely useful in reconstructing ancestral states of human rhythmic cognition (Merker et al., 2009; Ravignani et al., in press). Howler monkeys (genus *Alouatta*) also engage in group chorusing behavior (Sekulic, 1982), although the overall outcome is not synchrony and shows no obvious temporal coordination. An interesting question for future research is whether howler choruses simply sound asynchronous as an outcome of random timing of call onsets, or whether they exactly reach the statistical steady state predicted by physical models (see **Figure 4** and Strogatz and Stewart, 1993).

### ISOCHRONY AND GROUP BONDING

What about humans? An overview of chorusing behavior in primates would be incomplete without discussion of the evolutionary function of rhythmic abilities in our own species. One functional hypothesis of human pulse perception and entrainment (i.e., the ability to extract a regular pulse from music and adjust some aspect of behavior to it) comes from the work of Bjorn Merker. Merker et al. (2009) have extended the beacon hypothesis to our primate ancestors, suggesting that synchronous chorusing in multi-male displays served to better attract migrating females, increasing their chances of settling and ultimately reproducing with a particular group of males (Merker, 2000). By this hypothesis, the quality of synchrony between chorusing males may have influenced female choice in several ways. First, synchronous vocalization results in higher-power composite signals that travel farther with greater intensity, thus having more potential to attract females. Second, increased intensity may have served as an indication of the resource richness of a territory held by a male group, as more resources would support more males and perhaps also allow more time and energy for display. And third, the quality of synchrony may have indicated something about the capacity of a particular group for cooperation, with further implications for resource acquisition and territorial defense (Merker, 2000). To the extent that these factors actually did affect the choice to settle by migrating females, there would have been sexual selection on males to develop entrainment skills. In this context, the function of pulse perception and entrainment was originally to attract females.

On a mechanistic level, Merker et al. (2009) focus on the fact that the perception of a temporally regular pulse allows for accurate prediction of events in time, and thus may have arisen as a means of achieving synchrony. This mechanistic part of Merker’s hypothesis was recently tested in a speech synchronization experiment (Bowling et al., 2013). In this study, participants were asked to read short nonsense-word sentences aloud in two conditions: alone or together with a partner. Comparisons of speech timing between these two conditions showed that the durational intervals between words were significantly more regular in the together condition than in the alone condition, suggesting that synchronous vocal production is indeed a plausible mechanism driving isochronous signal production. Further, the participants in this experiment were always paired with a member of the same gender, allowing us to examine whether there are differences in synchronization ability between males and females. If human pulse perception and entrainment was shaped primarily by a female preference for males with good synchronization abilities (as Merker’s hypothesis suggests), we might expect males to exhibit better synchronization abilities than females^2^. However, no significant differences between male–male and female–female pairs were observed, either in quality of synchronization (measured as the average onset difference between participants’ word onsets), or the speed with which synchrony was achieved (measured as the number of attempts required to achieve an average onset difference below 40 ms). These results suggest that men and women are roughly equal in their vocal entrainment abilities (reflecting the observation that there are no obvious differences in the timing abilities of male and female vocalists). While this evidence certainly does not rule out a role for sexual selection in the evolution of human pulse perception and entrainment, it provides no support for an account based solely on female choice for synchronizing males^3^.

A second functional account of human pulse perception and entrainment comes from work examining the influence of interpersonal synchrony on social behavior (McNeill, 1997; Hagen and Bryant, 2003; Hagen and Hammerstein, 2009; Wiltermuth and Heath, 2009). Over the past 30 years, evidence has accumulated that engaging in interpersonal synchrony leads to a number of important changes in other social behaviors. Synchronized singing, for example, results in increased trust and cooperation (Anshel and Kipper, 1988), as does synchronization of other complex actions, such as walking and bimanual object manipulation (Wiltermuth and Heath, 2009). Synchronization has also been demonstrated to increase interpersonal affiliation (Hove and Risen, 2009; Miles et al., 2009), as well as the probability of engaging in helping behavior (Kirschner and Tomasello, 2010). In interpreting this evidence it is important to note that despite our current situation in the age of the personal music player (Walkmans^TM^, iPods^TM^, etc.), the vast majority of our species’ past experience with music has occurred solely in social contexts, in which our propensity for pulse perception and entrainment and its associated interpersonal synchrony could have had immediate prosocial consequences. Integrating these results and observations leads to what might be called the synchrony and sociality hypothesis for human pulse perception and entrainment. This hypothesis states that our behavioral tendency to move to music is the signature of an evolutionary process in which prosocial consequences of interpersonal synchrony conferred a fitness advantage on individuals in groups that practiced music (Bowling et al., 2013).

## HYPOTHESES AND FUTURE RESEARCH

### MAPPING INDIVIDUAL TIMING TO CHORUSING DYNAMICS

We have seen how different evolutionary pressures to lead, follow, hide, or cooperate in signaling can lead to various subtypes of chorusing, such as synchrony and antiphony. What about the relationship between specific temporal patterns produced at the individual level and specific temporal patterns at the chorusing level? How do individual patterns map onto to chorus patterns? Given the definitional framework outlined in **Figure 2**, the next step will be to examine these mappings (see [Ravignani, 2014; Ravignani et al., 2014]). Other questions include, how do cooperation and competition map onto chorusing behavior? Do these different types of interaction consistently lead to specific types of chorusing? And how do small perturbations in strategy affect chorusing behavior? For example, the switch from reacting to a conspecific’s call offset, instead of its onset, could result in a change from synchrony into a continuous stream of sound.

### THE EVOLVING SIGNAL TIMING HYPOTHESIS

How can we relate chorusing behavior systematically to evolution? One approach is to integrate findings from previous studies of animal communication with future research in comparative bio-musicology. Often, closely related species show similar chorusing behavior. This makes sense, as closely related species typically share a common ancestor that presumably had a similar brain and social system, and thus potentially similar cognitive capacities for supporting rhythm perception and production. Building on this, we can hypothesize that animals should tend to exhibit within-clade homogeneity and between-group continuity in their chorusing behavior, which in turn should influence individual timing abilities. More specifically, we propose the following *Evolving Signal Timing* hypothesis, which consists of two principal components:

(i) The more closely two species are related, the more their respective chorusing repertoires will occupy contiguous, possibly overlapping, areas in the hierarchy of possible chorusing rhythms (see **Figure 2B** and Ravignani et al., 2014). Importantly, this hypothesis predicts that the chorusing behavior of related species will be similar, but does not necessarily predict that the chorusing behavior of very distantly related species will be dissimilar (e.g., because of convergent evolution). We know, for example, that despite being distantly related, frogs and crickets exhibit similar chorusing repertoires. Hence common ancestry would constitute a sufficient (although not necessary) condition for similar chorusing behavior.

(ii) Interactive behavior shapes individual timing abilities, i.e., chorusing shapes individual timing. Using the definitions introduced in “Definitions” section, we suggest that individual timing abilities (**Figure 2A**) may have evolved under pressure to produce specific types of choruses (**Figure 2B**).

We can hence formulate the Evolving Signal Timing hypothesis in full: Closely related *chorusing* species will have similar individual timing abilities, whereas closely related species that do not chorus can, but will not necessarily, have similar individual timing abilities. This hypothesis is built on two commonplace assumptions in evolutionary biology: cognitive/behavioral continuity due to common ancestry (point i above); and ontogenetic/evolutionary plasticity of biological organisms (point ii).

Taking two different species as an example, a number of possibilities arise regarding the relationship between their respective chorusing behaviors. According to the Evolving Signal Timing hypothesis, if the species are closely related and both chorus, they should have comparable individual timing abilities. If two species are closely related, but only one exhibits chorusing behavior, timing abilities might still be comparable. Their last common ancestor could have exhibited chorusing, which was then subsequently lost in one daughter species but retained in the other. For distantly related species, the prediction would be that individual timing abilities will tend to differ, unless one of those species experienced similar evolutionary pressures on chorusing (where convergence is expected). For closely related species in which neither exhibits chorusing behavior, no predictions can be made.

The Evolving Signal Timing hypothesis differs from and extends previous hypotheses (e.g., Patel, 2006; Merchant and Honing, 2013; Patel and Iversen, 2014) on the evolution of rhythmic abilities in two related fundamental respects. First, it does not take a single species (e.g., humans) as its reference point, instead it provides predictions that apply across pairs or groups of species. Comparisons between humans and other animals are, for us, just one of a large set of interesting possible comparisons. Second, it makes predictions about rhythmic abilities beyond the traditional scope of entrainment to an isochronous pulse (Patel, 2006) or interval-based estimation (Merchant and Honing, 2013).

The Evolving Signal Timing hypothesis is in principle compatible with previous related hypotheses, such as the Gradual Audiomotor Evolution (Merchant and Honing, 2013) or the Action Simulation for Auditory Prediction (Patel and Iversen, 2014) hypotheses. In addition, it provides theoretical tools and experimental avenues to investigate – via comparative research in marine mammal communication – why sea lions are capable of entrainment despite (apparently) being incapable of vocal learning (Cook et al., 2013), a finding yet to be explained by any theoretical framework.

## DISCUSSION AND CONCLUSION

### DISCUSSION

In this paper we examined chorusing behavior in non-human animals, with the intent of informing research on the evolutionary function(s) of rhythm in humans. We reviewed how several species, often neglected in discussions of rhythmic cognition, interact in their natural environments to produce complex temporal patterns, and we discussed how individual timing abilities are shaped by chorusing. We have emphasized the potential importance of chorusing behavior in insects and anurans in rhythm research. While it seems unlikely that the nervous system of a katydid or tropical tree frog will provide insights into the neural mechanisms that govern rhythm perception and production in humans, we have argued that careful study of insect and anuran behavior can nonetheless teach us about selective pressures that shape rhythmic behavior in general. Despite being capable of entraining in choruses that exhibit many phenomena typical of human rhythms (e.g., isochrony, synchrony, antiphony), these species have too rarely been directly compared to humans in a systematic fashion.

Such comparisons across a diverse range of taxa can provide insights into the selective pressures that shape the evolution of rhythmic behavior, raising the possibility that similar pressures played an important role in shaping the rhythmic abilities of our proto-musical ancestors. We provided a definitional framework for a comparative approach to rhythm perception and production, with the hope that future comparisons take advantage of this system to highlight important similarities and differences across species. In light of the definitions and broad comparative review provided here, additional questions and testable hypothesis can be more clearly formulated.

The Evolving Signal Timing hypothesis we propose provides a framework for investigating homologies, analogies and differences in rhythmic abilities across and within species. It will hopefully enable experimental researchers to fill in a matrix of pair-wise relations between species, quantifying the similarities and differences in their individual rhythmic repertoires.

### CONCLUSION

Bio-musicology can profitably adopt a comparative approach based on multiple components of music, and fruitful comparisons have already been drawn between melodic aspects of music and spectral properties of animal calls. Temporal factors in animal communication have received less attention in relation to musical rhythm. In this paper we have extended the scope of comparative approaches to the biology of rhythm. After providing general definitions, we illustrated how rhythm, synchrony, and chorusing relate to one another through basic biological and physical principles. Moreover, augmenting the classical approach to rhythm investigated in single agents, we suggested how *group* chorusing behavior can shape individual timing abilities. As in human music and dance, chorusing interactions in animals both influence individual timing, and are influenced by it. Accordingly, we argued that chorusing behavior constitutes an important aspect of the comparative framework for investigating rhythm, in particular its adaptive function.

In conclusion, although mechanistic and functional questions are always linked in biological systems, they typically require different levels of explanation, and different types of data. Biomusicology can gain a deeper understanding of the evolution of rhythm by going beyond the vertebrate species more typically considered (mostly bird and mammal species), and exploring a broader range of taxa (orthopterans, fireflies, frogs, etc.). The array of living organisms exhibiting chorusing behavior, and the variety of evolutionary pressures acting on chorusing can provide crucial insights into the adaptive function(s) of rhythmic behavior in our “proto-musical” primate ancestors, and in turn inform our understanding of the biology and evolution of rhythm in human music and language.

## FINANCIAL DISCLOSURE

Andrea Ravignani was supported by ERC Advanced Grant SOMACCA (#230604) to W. Tecumseh Fitch. Andrea Ravignani acknowledges additional support of the University of Vienna (KWA grant). Daniel L. Bowling was supported by a grant from the University of Vienna to W. Tecumseh Fitch. The funders had no role in study design, data collection and analysis, decision to publish, or preparation of the manuscript.

## AUTHOR CONTRIBUTIONS

All authors wrote, edited, and approved the manuscript.

## Conflict of Interest Statement

The authors declare that the research was conducted in the absence of any commercial or financial relationships that could be construed as a potential conflict of interest.

## Footnotes

1 Typically, real world patterns are quasiperiodic rather than periodic, meaning they are nearly but not perfectly regular, we nevertheless use the term periodic throughout this paper for simplicity. Also note that the distinction between periodic and aperiodic typically depends upon the duration of the analysis window, e.g., a given pattern might be aperiodic over a period of 10 s, but repeat thereafter. Few temporal patterns observed in animal communication are truly aperiodic, with certain features of human speech providing possible exceptions.

2 This would be particularly true in polygynous mating systems. In a monogamous mating system, which Merker argues may have applied to our early hominid ancestors (Merker et al., 2009), sexual selection can be bi-directional and thus lead to the development of a trait in both sexes. Under these conditions, if both sexes favored mates with good synchronization abilities, one would predict more balanced synchronization abilities between the sexes. This possibility, however, represents a considerable departure from the logic of Merker’s beacon hypothesis.

3 One way in which Merker’s hypothesis on the evolution of human pulse perception and entrainment remains viable despite the evidence presented in Bowling et al. (2013) concerns the genetic basis of synchronization abilities (unknown at this point). Female choice for synchronizing males could have resulted in the transmission of the relevant genetic underpinnings to both sexes. Experimental selection of a trait in one sex often leads to a response of the homologous trait, if any, in the opposite sex (Lande, 1980; Bonduriansky, 2007).
